# Supplementary material for: Dynamic transcriptome and chromatin architecture in granulosa cells during chicken folliculogenesis
Source: Nat Commun. 2022 Jan 10;13:131. doi: 10.1038/s41467-021-27800-9 (PMC8748434; doi:10.1038/s41467-021-27800-9)
Supplement: Supplementary file 1 — Supplementary Information [file 41467_2021_27800_MOESM1_ESM.pdf]

Supplementary Information

Supplementary Information Table of Contents

Supplementary Figs. 1-12

Supplementary Fig. 1 Expression profiles of chicken granulosa cells during folliculogenesis.

Supplementary Fig. 2 Distinct markers of chicken granulosa cells (GCs).

Supplementary Fig. 3 The characterization of Hi-C contacts in six samples.

Supplementary Fig. 4 The feature of compartment A/B in chicken granulosa cells.

Supplementary Fig. 5 Compartment switching in chicken granulosa cells during folliculogenesis.

Supplementary Fig. 6 The chromatin accessibility of chicken granulosa cells during folliculogenesis.

Supplementary Fig. 7 Characteristics of TADs in chicken granulosa cells.

Supplementary Fig. 8 Comparison of TAD intactness between developmental stages.

Supplementary Fig. 9 Stage-specific boundaries associated with changes of gene expression in granulosa cells during folliculogenesis.

Supplementary Fig. 10 Changes in the *D*-score of consensus TADs during folliculogenesis.

Supplementary Fig. 11 Basic features of PEIs and stage-specific changes in PEIs.

Supplementary Fig. 12 Examples of genome regions containing genes with different number of PEIs and convergent CTCF-CTCF loops.

Supplementary Tables. 1-4

Supplementary Table 1 Hi-C sequencing data quality.

Supplementary Table 2 Summary of Hi-C data.

Supplementary Table 3 The resolution of Hi-C contacts of each sample.

Supplementary Table 4 The resolution of Hi-C contacts with merged biological replicates in each stage.

Supplementary Figures

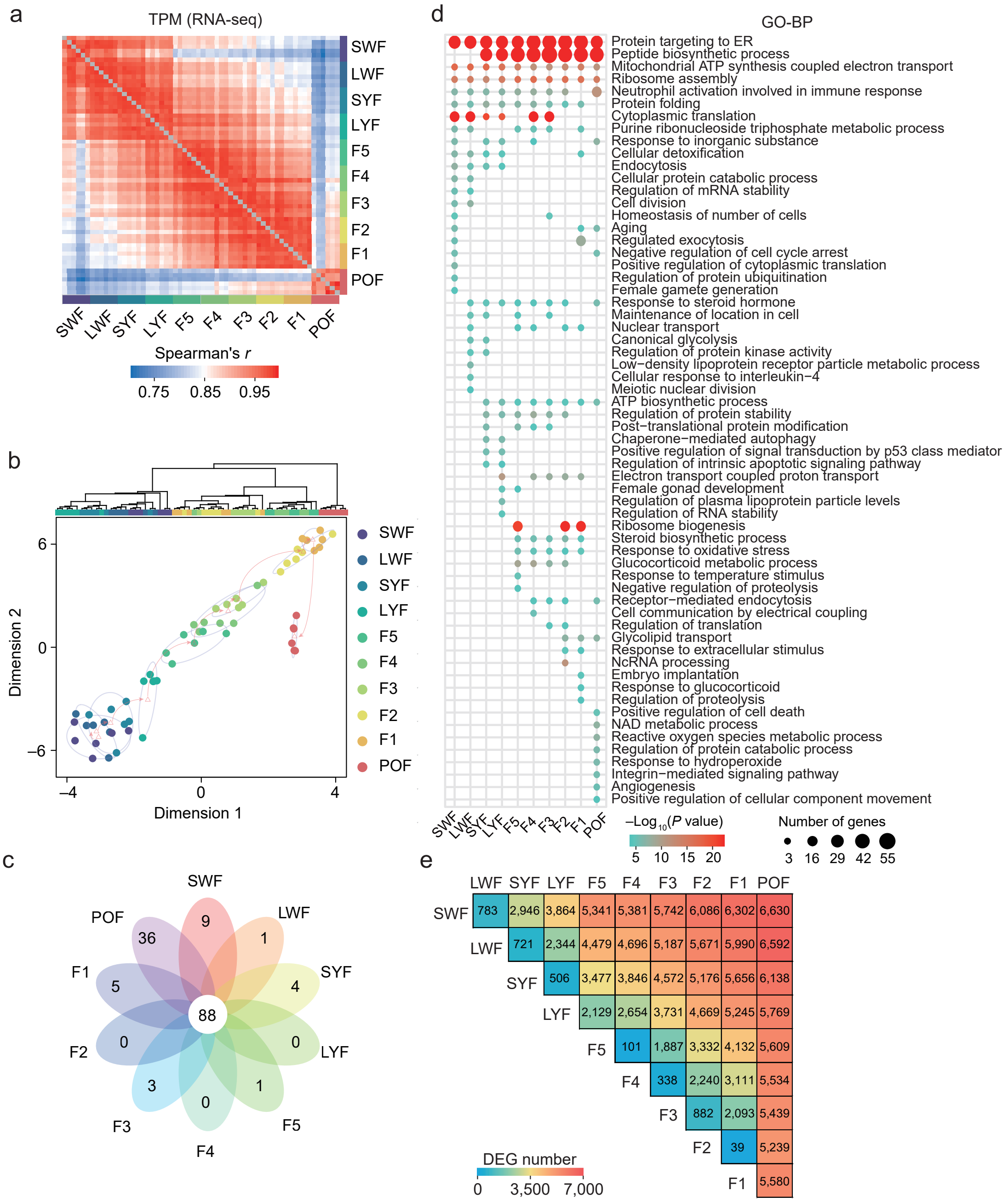

Supplementary Fig. 1 Expression profiles of chicken granulosa cells during folliculogenesis. a Spearman's *r* heatmap for gene expression profiles of the 60 samples. **b** The unsupervised hierarchical clustering (top panel) and t-Distributed Stochastic Neighbor Embedding analysis (t-SNE, bottom panel) of gene expression profiles for all samples. Pink triangles indicate centers of the minimum volume ellipse. **c** Flower plot of the top 1% most highly expressed genes (n = 168) at each stage. The peripheral numbers

represent the counts of stage-specific genes, and the central number represents the number of co-expressed genes across all stages. **d** The top 20 significantly enriched Gene Ontology-Biological Process (GO-BP) terms of the top 1% most highly expressed genes at each stage. **e** Heatmap of differentially expressed gene (DEG) numbers in pairwise comparisons among the ten stages. DEGs were identified using the threshold of $|\log_2 FC| > 1$ and false discovery rate (FDR) < 0.05. Source data are provided as a Source Data file.

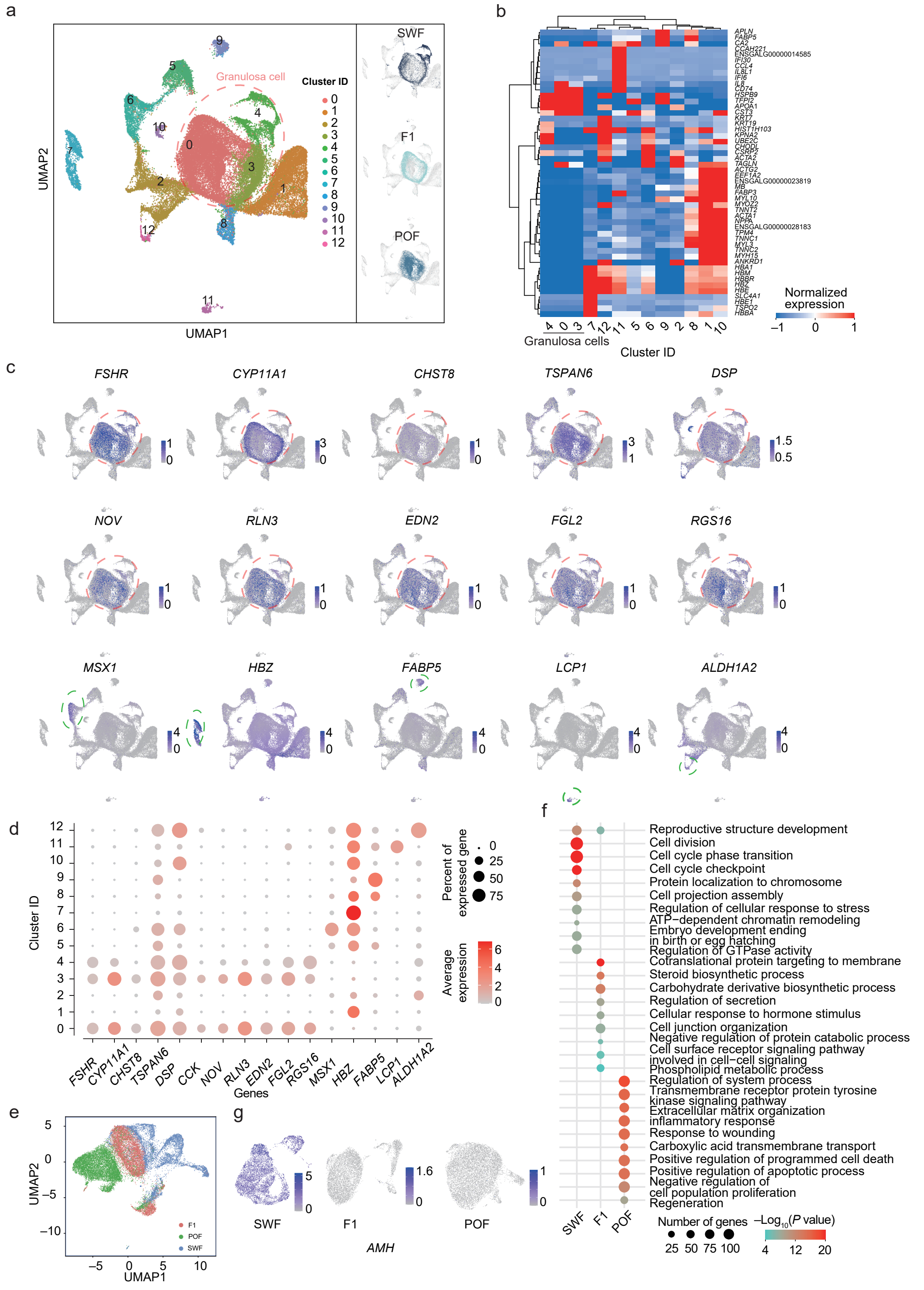

Supplementary Fig. 2 Distinct markers of chicken granulosa cells (GCs). a Left: UMAP revealing 13 specific clusters representing the chicken heart cell and GC types. The red dashed lines give the boundaries of the GC clusters of interest. Right: UMAP plots showing the granulosa cell distribution for SWF, F1, and POF. **b** Heatmap and hierarchical

clustering based on the expression of the top 50 most variable genes. **c** UMAPs showing expression of ten selected marker genes specifically expressed in GCs (*FSHR*, *CYP11A1*, *CHST8*, *TSPAN6*, *DSP*, *NOV*, *RLN3*, *EDN2*, *FGL2*, and *RGS16*) while five were identified in chicken heart cells (*MSX1*, *HBZ*, *FABP5*, *LCP1,* and *ALDH1A2*). The red and green dashed lines indicate the boundaries of the GC and heart cell clusters of interest. **d** Gene expression profiles of selected markers for GC and other cell clusters. **e** UMAP reveals the GCs identified during three follicle growth stages. **f** Functional enrichment analysis for DEGs in GCs among developmental stages. **g** UMAP expression of *AMH* during the three follicle growth stages. Source data are provided as a Source Data file.

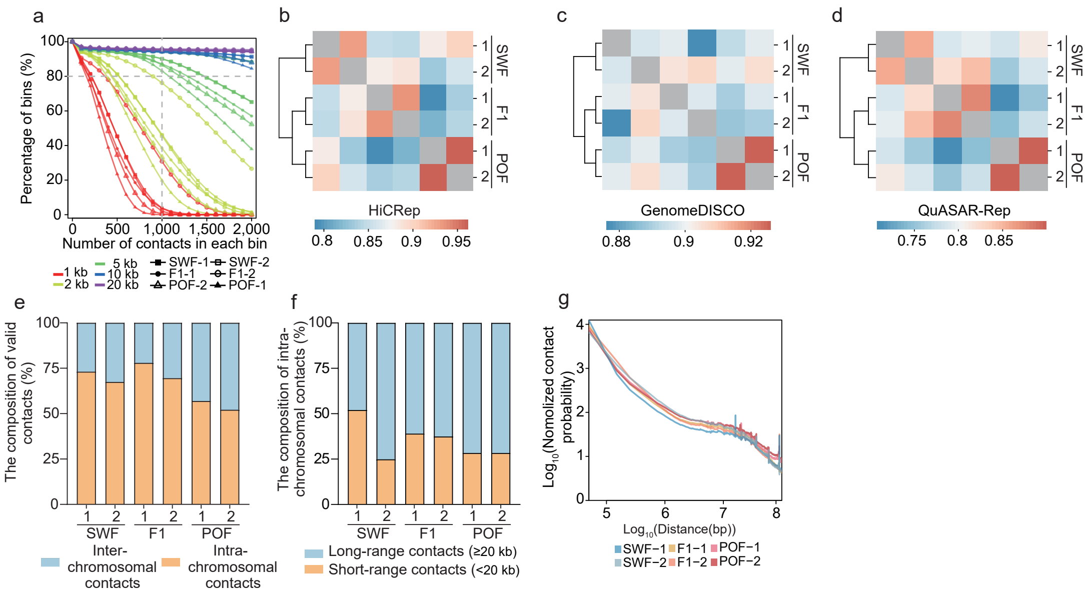

Supplementary Fig. 3 The characterization of Hi-C contacts in six samples. a Resolution estimation of intrachromosomal contact matrices. The map resolution is defined as the smallest bin size where 80% of the bins contain at least 1,000 reads to allow for the clear observation of local features. **b-d** Heatmaps of the HiCRep (**b**), GenomeDISCO (**c**), and QuASAR-Rep (**d**) correlation coefficients of the six samples. **e** The proportion of intrachromosomal and inter-chromosomal contacts in the valid contacts. **f** The proportion of long-range (≥ 20 kb) and short-range contacts (< 20 kb) in the intrachromosomal contacts. **g** The dependence of the contact probability on the genomic distance averaged over all chromosomes. Source data are provided as a Source Data file.

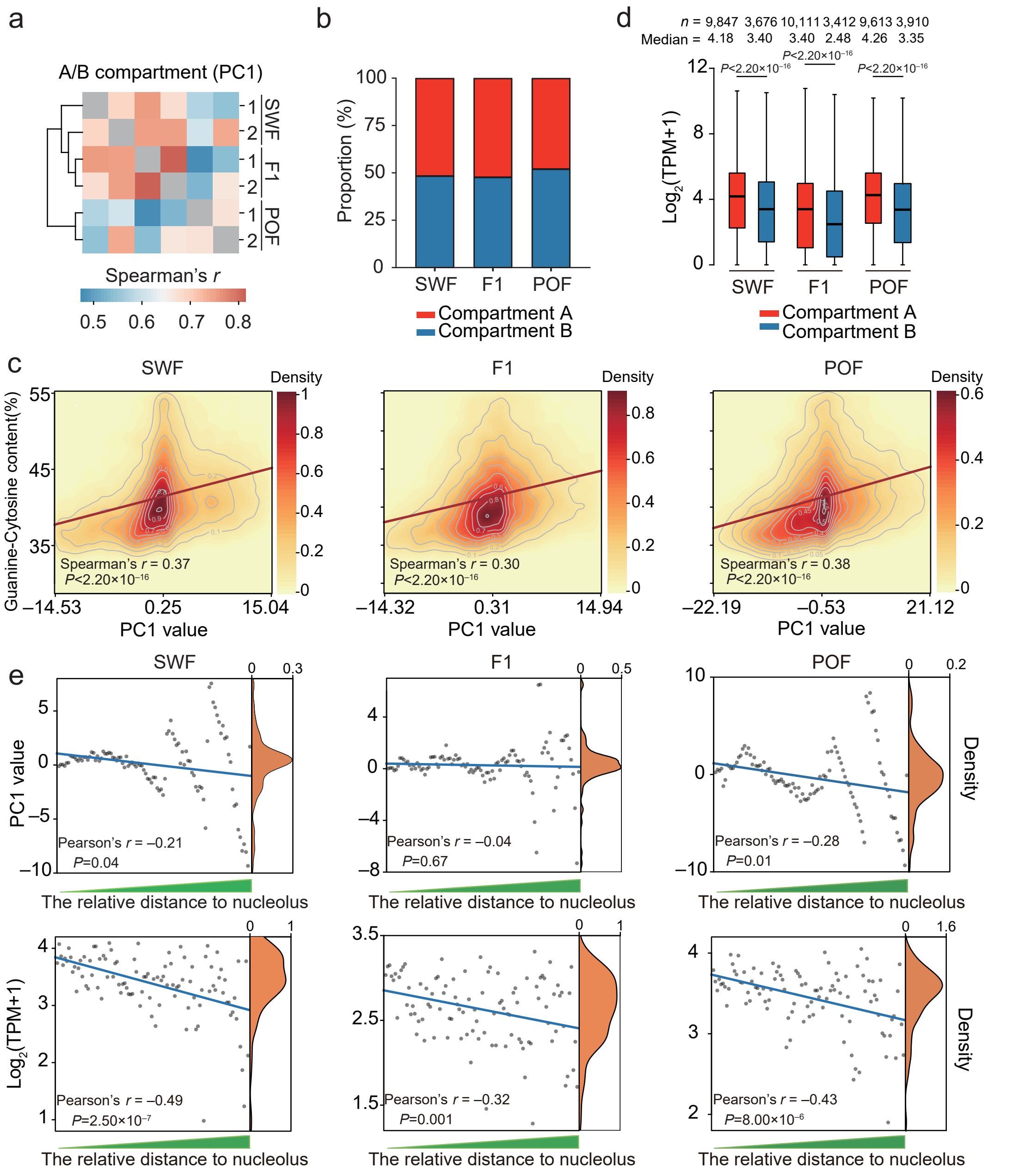

Supplementary Fig. 4 The feature of compartment A/B in chicken granulosa cells. a Spearman's *r* heatmap of PC1 values for the six samples at 20 kb resolution. **b** Proportion of A (~49.62%) and B (~50.38%) compartments in the chicken genome across granulosa cell development. **c** Intensity scatter plots showing the correlation between PC1 values and Guanine-Cytosine content. **d** Gene expression ($\log_2$(TPM+1)) in A or B compartments. In the boxplot, the internal line indicates the median, the box limits indicate the upper and lower quartiles and the whiskers extend to 1.5 IQR from the quartiles. **e** The Pearson's *r* between the relative nuclear radius and the PC1 value (top panels) or gene expression

(log_2(TPM+1)) (bottom panels). The density plots are shown on the right. *P* values in the **c** and **e** were calculated using two-sided hypothesis testing. *P* values in the **d** were calculated using two-sided Wilcoxon rank-sum test. Source data are provided as a Source Data file.

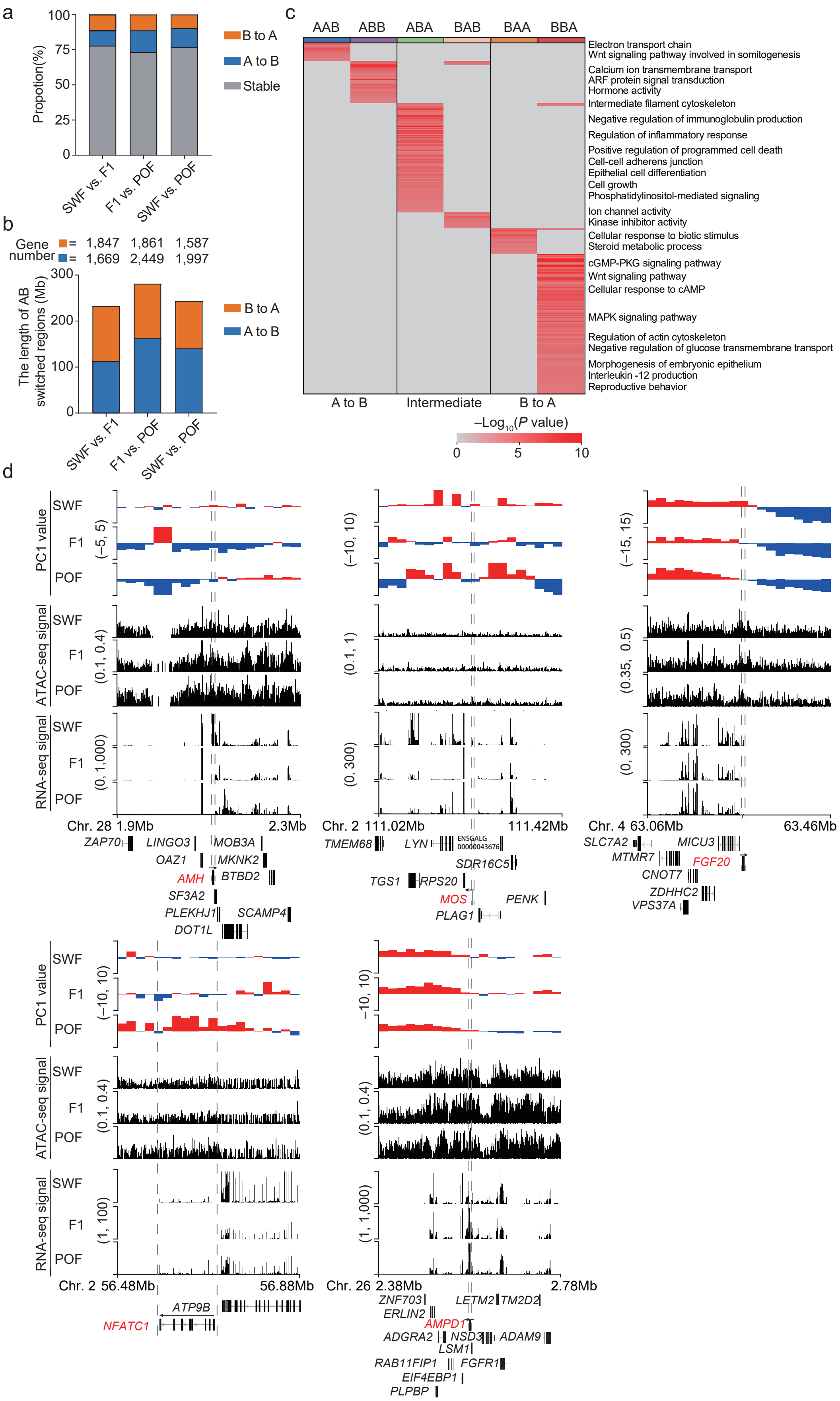

Supplementary Fig. 5 Compartment switching in chicken granulosa cells during

folliculogenesis. **a** Genomic proportion of stable or switched compartments. **b** The accumulative lengths of the switched compartments. Gene numbers within each type of region are indicated above the bar plot. **c** Significantly enriched GO-BP terms for genes located in the six types of A/B compartment transitions. **d** Five representative functional genes (red) undergoing compartment switching during folliculogenesis, including *AMH*, *MOS*, *FGF20*, *NFATC1,* and *AMPD1*. The dashed line boxes indicate the chromosomal locations of the genes. The tracks show the compartment (top panels), ATAC-seq signal (middle panels), and gene expression (bottom panels) features within 400 kb. Gene structures are indicated below the tracks. The black arrows indicate the direction of the gene transcription. Source data are provided as a Source Data file.

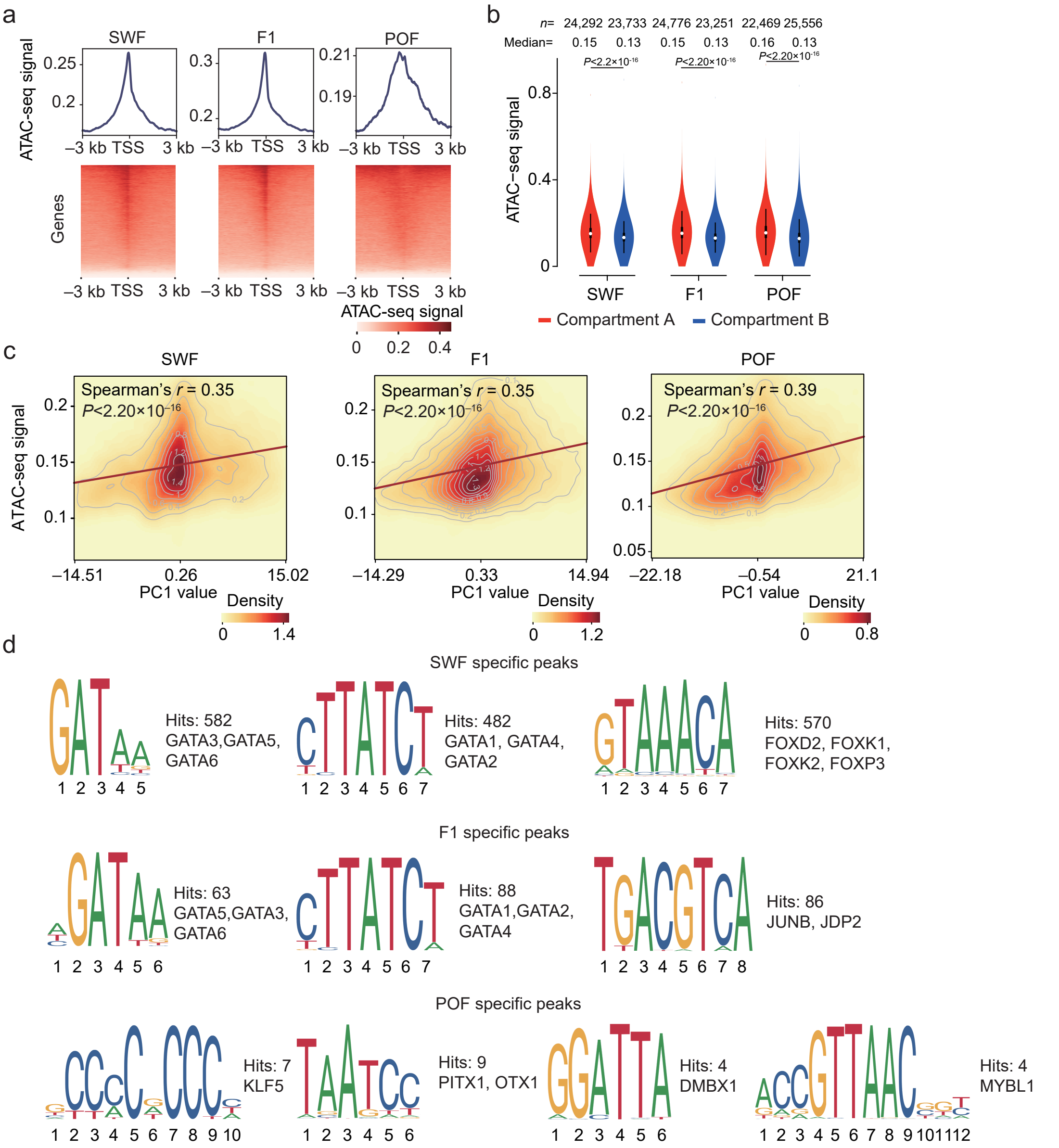

Supplementary Fig. 6 The chromatin accessibility of chicken granulosa cells during folliculogenesis. **a** ATAC-seq signals are enriched at transcriptional start sites (TSSs). Top: frequency distribution of ATAC-seq signals at TSS regions. Bottom: heatmaps of ATAC-seq signals at TSS regions, sorted by signal intensity. **b** Significant difference in the enrichment of ATAC-seq signals in A/B compartments. The white dot indicates the median, the bold line indicates the upper and lower quartiles and the whiskers extend to 1.5 IQR from the quartiles. **c** Intensity scatter plots showing the correlation between PC1 value (A/B compartments) and ATAC-seq signals. **d** The most significantly enriched motifs identified within SWF-, F1-, and POF-specific ATAC-seq peaks using DREME. *P* values in the **b** were calculated using two-sided Wilcoxon rank-sum test. *P* values in the **c** were calculated using

two-sided hypothesis testing. Source data are provided as a Source Data file.

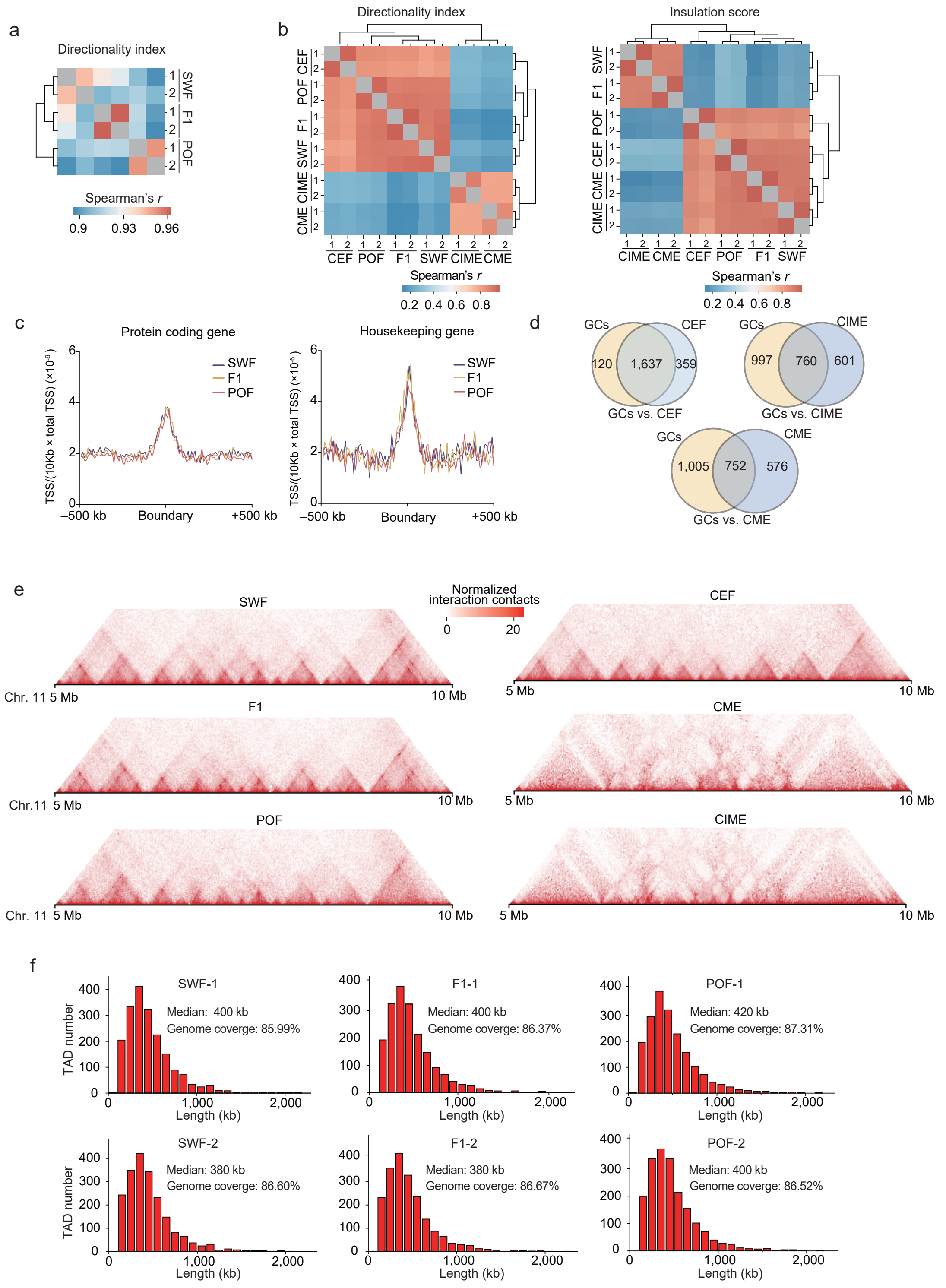

Supplementary Fig. 7 Characteristics of TADs in chicken granulosa cells. a Spearman's *r* heatmap of the directionality index (DI) among the six samples, showing a high reproducibility within biological replicates (Spearman's *r* > 0.94). **b** Spearman's *r* heatmap of the DI (left panel) and IS (right panel) for GCs and other chicken cell types. CIME: chicken adult immature erythrocytes, CME: chicken adult mature erythrocytes, CEF:

chicken embryonic fibroblasts. **c** Enrichment of protein-coding genes and housekeeping genes in TAD boundaries. **d** Overlap of TAD boundaries between different chicken cell types. **e** Hi-C contact maps of a representative genomic region (Chr.11: 5–10 Mb) showing different TAD structures across different chicken cell types. **f** Frequency distribution histograms of TAD size in six samples. Source data are provided as a Source Data file.

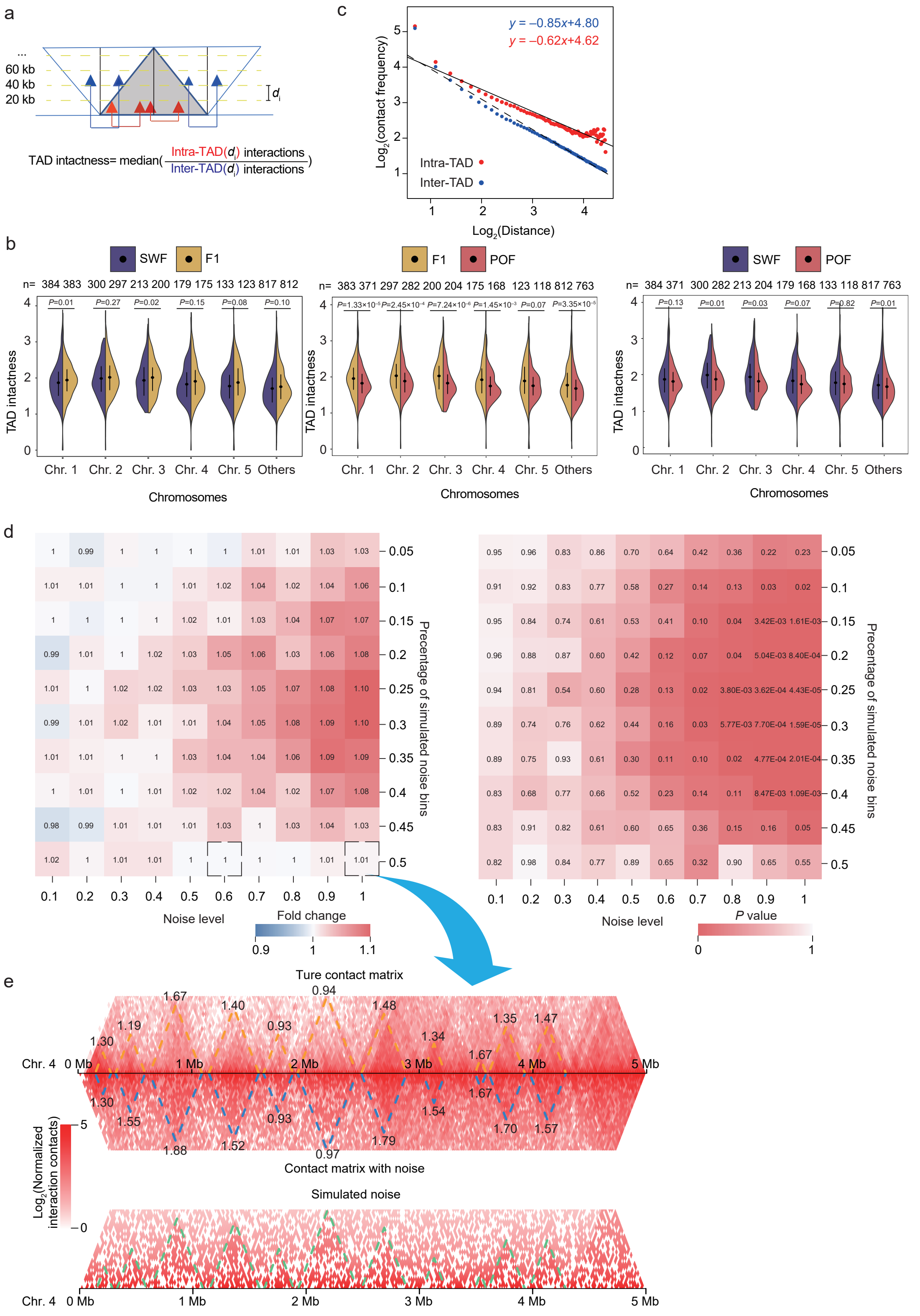

Supplementary Fig. 8. Comparison of TAD intactness between developmental stages. a Illustration of TAD intactness calculation. **b** Comparison of TAD intactness between stages. The chromosomes 1 to 5 that have more than 100 TADs are displayed separately, while TADs in other chromosomes are grouped and displayed as “Others”. In

the violin plot, black dot and line indicate mean ± S.D. *P* values were calculated using two-sided Wilcoxon rank-sum test. **c** The dependence of the contact frequency on the genomic distance for intra- and inter-TAD over chromosome 4 (contact matrices are derived from the first replicate of SWF). A linear regression model was applied to fit each scatter plot, and the intercept and coefficient parameters were estimated. **d** Simulation results for changes of TAD intactness with variable noise levels. To investigate the robustness of the TAD intactness measurement, we generated the simulated contact matrix by randomly adding signal noises to different proportions (from 5% to 50%) of the contact matrix at different noise signal levels (from 10% to 100% of the average contact frequency at each distance in original contact matrix). For noise signal levels, we distinguish intra- and inter-TAD 2D bins by using different averages based on the distinct estimated power-law decay in (**c**). We found that TAD intactness was highly robust across variable noise levels. Even at noise levels as high as 60% for 50% of the contact matrix, the TAD intactness between the original and simulated contact matrix is approximately equal (fold change of intactness = 1, *P* = 0.65, two-sided Wilcoxon rank-sum test). **e** Example of the original contact matrix (chromosome 4: 0-5 Mb) with the randomly added signal noise (50% proportion with 100% noise level). TADs are marked with dashed lines and their intactness values are also listed. Source data are provided as a Source Data file.

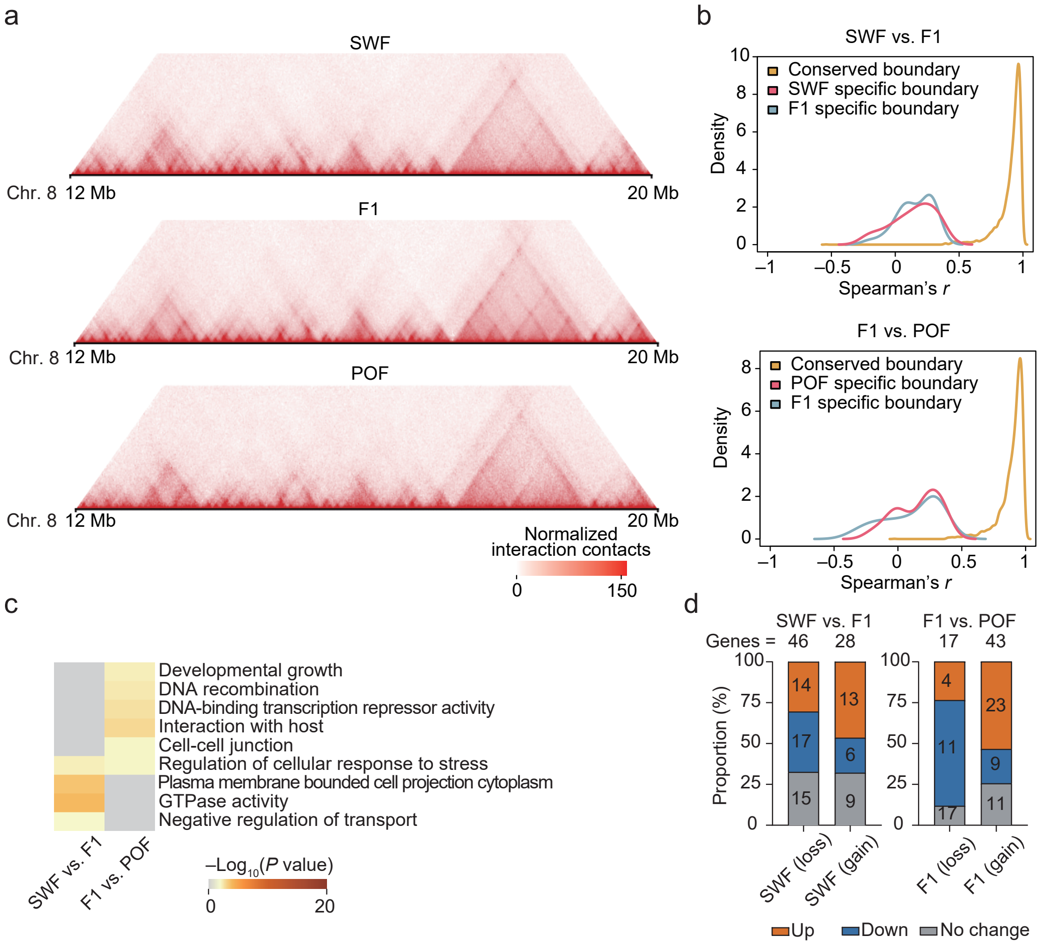

Supplementary Fig. 9 Stage-specific boundaries associated with changes of gene expression in granulosa cells during folliculogenesis. a Hi-C contact maps in a region of chromosome 8 (12–20 Mb) showing distinct TAD structures at the three stages. **b** Density plot of the Spearman's *r* of DI values for TAD boundary regions (± 200 kb) between adjacent stages. **c** The significantly enriched GO-BP terms for genes located at stage-specific boundaries. **d** Proportion of shifted boundaries containing upregulated, downregulated, and stably expressed genes. Boundaries were classified based on whether they were lost or gained during folliculogenesis. Source data are provided as a Source Data file.

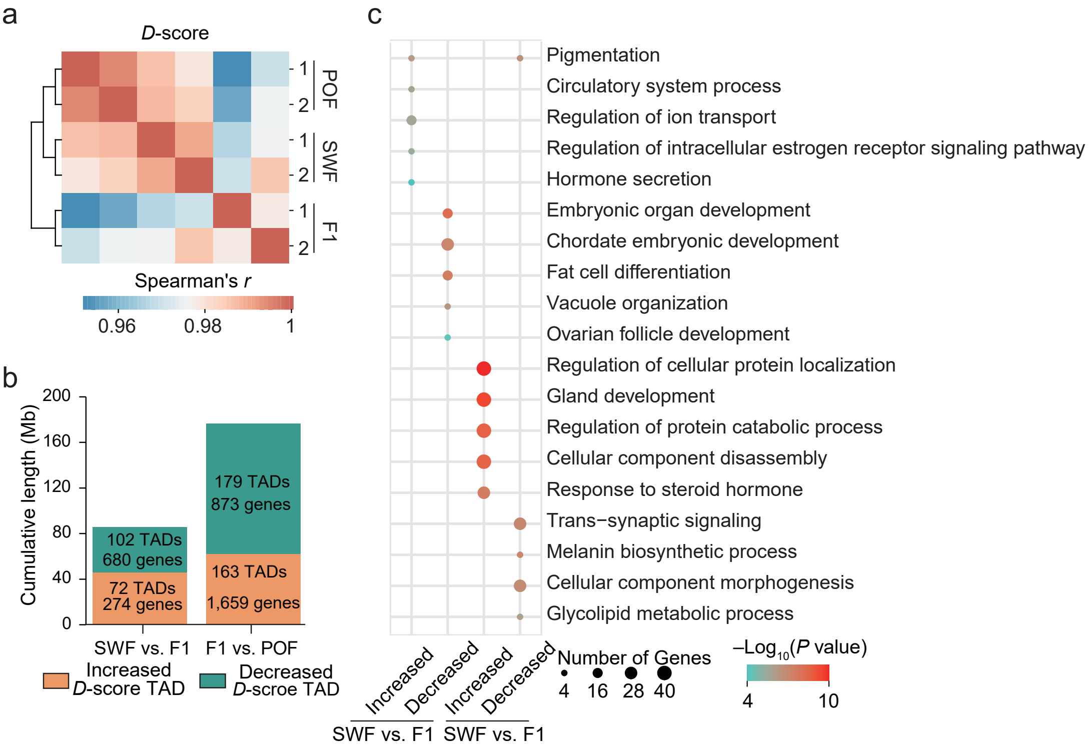

Supplementary Fig. 10 Changes in the *D*-score of consensus TADs during folliculogenesis. a Spearman's *r* heatmap of the *D*-score in consensus TADs among the six samples. **b** Accumulative lengths of consensus TADs with increased or decreased *D*-scores between adjacent stages. The numbers of TADs and genes are indicated on the bar plot. **c** Selected enriched GO-BP terms for genes in TADs with significant changes in their *D*-score between adjacent stages. Source data are provided as a Source Data file.

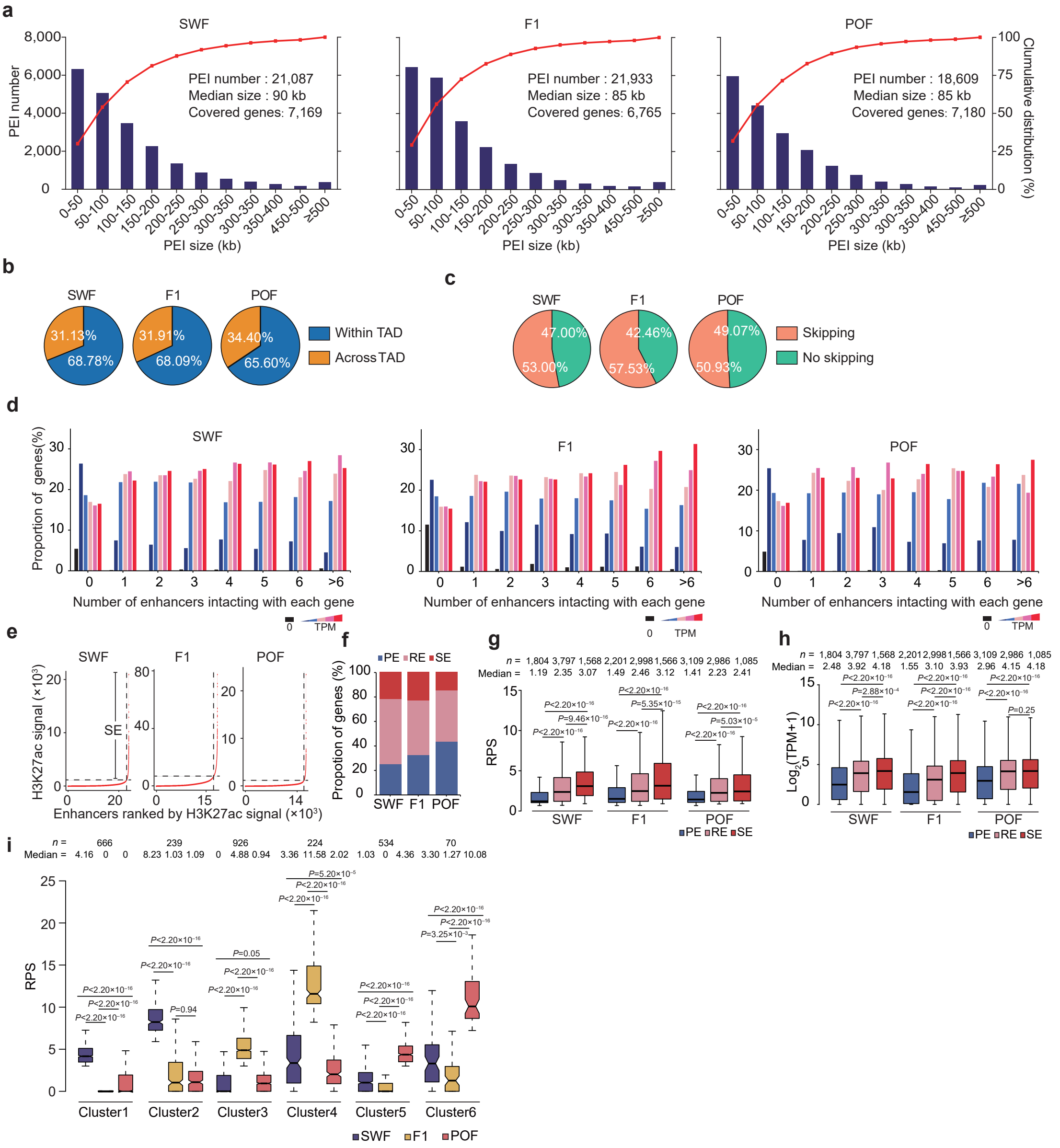

Supplementary Fig. 11 Basic features of PEIs and stage-specific changes in PEIs. a Size distribution of PEIs at each stage. The red lines represent the percentiles of cumulative distribution. **b** Proportion of PEIs located within or across TADs. **c** Ratio of enhancers interacting with the nearest promoters (no skipping) or skipping at least one promoter in each stage. **d** Proportion of genes from each expression category interacting with different number of enhancers. **e** Determination of super-enhancers (SEs) by ranking H3K27ac signals using the ROSE algorithm. **f** Proportion of PEI-associated genes interacting with super-enhancers (SEs), regular enhancers (REs), and poised enhancers (PEs) at each stage. **g** RPS values and **h** expression levels of genes interacting with SEs, REs, and PEs. **i** Comparison of RPS for genes in each cluster during folliculogenesis. For

panels **g**, **h**, and **i**, the internal line indicates the median, the box limits indicate the upper and lower quartiles and the whiskers extend to 1.5 IQR from the quartiles. *P* values in **g**, **h**, and **i** were calculated using two-sided Wilcoxon rank-sum test. Source data are provided as a Source Data file.

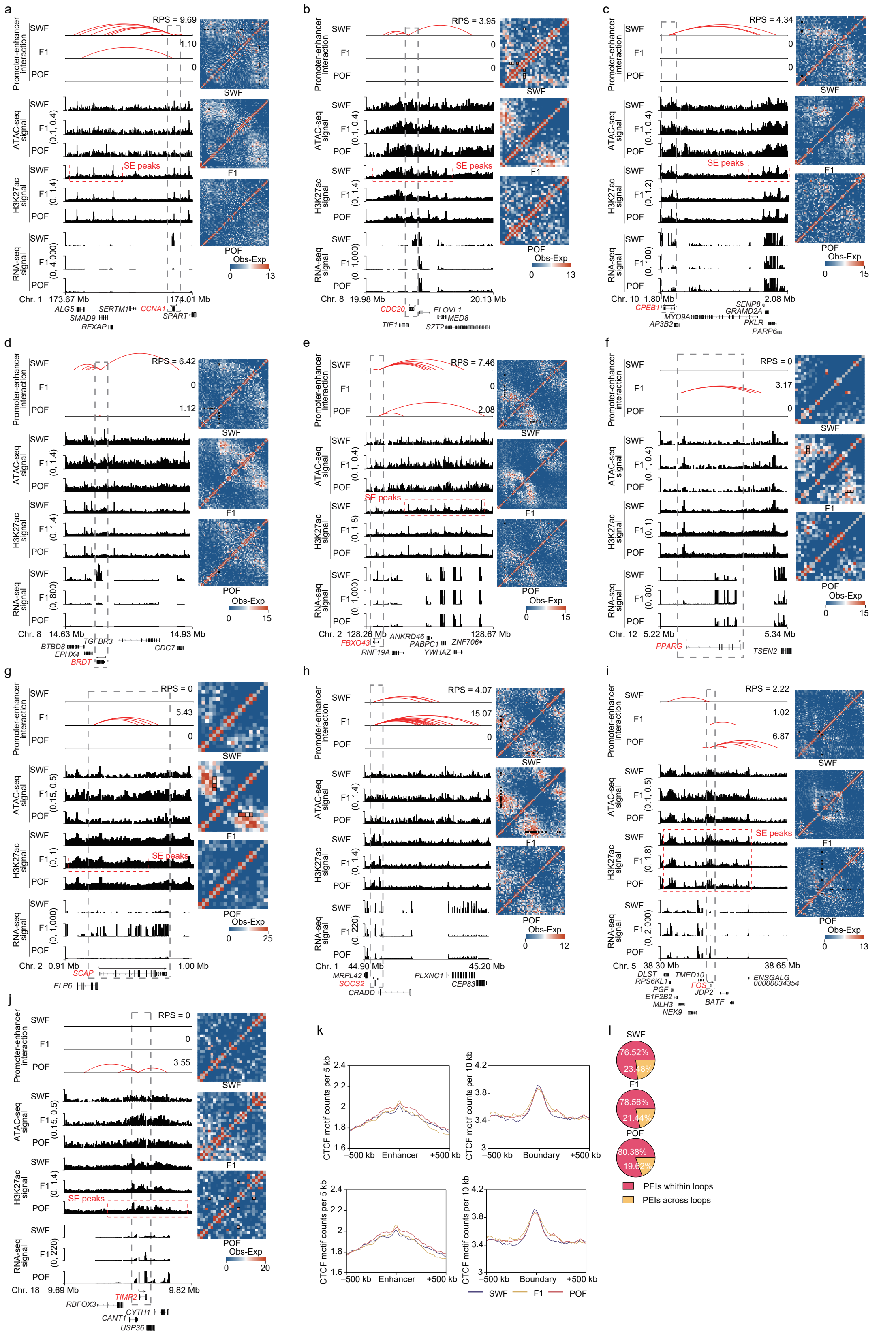

Supplementary Fig. 12 Examples of genome regions containing genes with different number of PEIs and convergent CTCF-CTCF loops. Including *CCNA1* (**a**), *CDC20* (**b**), *CPEB1* (**c**), *BRDT* (**d**), *FBXO43* (**e**), *PPARG* (**f**), *SCAP* (**g**), *SOCS2* (**h**), *FOS* (**i**), and *TIMP2*

(**j**). Left: schematics of PEIs (top) and tracks of ATAC-seq signals, H3K27ac signals, and expression levels. Gene structures are displayed below the tracks. Super-enhancer peaks (SE peaks) are indicated with red dashed line boxes on the tracks of the H3K27ac signals. The gray dashed line boxes indicate the chromosomal locations of the genes. Right: Hi-C contact maps of these genomic regions. **k** Enrichment of CTCF motifs at enhancer regions and TAD boundaries. **l** Proportion of PEIs within and across convergent CTCF-CTCF loops. For genes located within the CTCF-CTCF loops, the majority of their PEI events (76.52%, 78.56%, and 80.38% for SWF, F1, and POF, respectively) occur within loops (see **Method** and **Supplementary Data 5**). Source data are provided as a Source Data file.

Supplementary Tables

Supplementary Table 1 Hi-C sequencing data quality.

Sample name	Raw base (bp)	Clean base (bp)	Effective rate (%)	Error rate (%)	Q20 (%)	Q30 (%)	Guanine-Cytosine content (%)
SWF-1	236,260,070,100	234,930,481,800	99.44	0.03	97.03	92.23	41.49
SWF-2	176,580,601,200	174,473,787,600	98.81	0.03	96.46	90.64	42.19
F1-1	170,072,199,300	169,519,417,500	99.67	0.03	96.94	92.01	41.59
F1-2	185,606,851,500	185,129,025,000	99.74	0.03	96.6	91.47	41.89
POF-1	152,236,332,300	150,735,755,400	99.01	0.03	96.32	90.97	43.59
POF-2	182,614,821,000	180,983,092,800	99.11	0.03	96.22	90.66	42.70

Supplementary Table 2 Summary of Hi-C data.

Sample name	Sequenced read pairs (Mb)	Normal paired (Mb)	Chimeric paired (Mb)	Chimeric ambiguous (Mb)	Unmapped (Mb)	Alignable (Normal+Chimeric Paired) (Mb)	Unique reads (Mb)	PCR duplicates (Mb)	Intra-fragment reads (Mb)	Low mapping quality (<30) (Mb)	HiC validly aligned contacts (Mb)	Inter chromosomal (Mb)	Intra chromosomal (Mb)	Intra short range (<20 kb) (Mb)	Intra long range (>20 kb) (Mb)
SWF-1	783.10	434.64	310.93	27.60	9.94	745.57	369.83	371.30	21.07	33.05	315.70	84.78	230.92	120.17	110.75
SWF-2	581.58	231.88	304.32	38.36	7.02	536.20	397.23	130.25	1.40	34.08	361.75	117.65	244.09	60.72	183.37
F1-1	565.06	307.20	231.90	19.12	6.85	539.10	318.83	217.06	13.48	25.99	279.36	61.55	217.80	85.01	132.80
F1-2	905.32	352.10	468.76	71.85	12.61	820.86	628.69	187.94	3.75	54.46	570.48	173.75	396.73	148.55	248.18
POF-1	502.45	216.71	247.20	29.95	8.59	463.91	353.43	106.12	1.87	37.55	314.01	135.09	178.93	50.73	128.19
POF-2	603.28	291.12	271.43	29.30	11.42	562.56	448.29	110.13	2.28	44.66	401.35	192.13	209.22	59.44	149.78

Supplementary Table 3 The resolution of Hi-C contacts of each sample.

**Cis**													
**Sample name**	**1kb**	**2kb**	**5kb**	**10kb**	**20kb**	**30kb**	**40kb**	**50kb**	**60kb**	**70kb**	**80kb**	**90kb**	**100kb**
SWF-1	3.76%	46.31%	90.09%	94.54%	95.44%	95.87%	96.22%	96.44%	96.70%	96.90%	96.95%	97.12%	97.27%
SWF-2	2.05%	45.63%	90.12%	94.34%	95.28%	95.68%	96.01%	96.26%	96.52%	96.78%	96.86%	96.98%	97.08%
F1-1	3.81%	38.44%	87.17%	93.89%	95.20%	95.64%	95.99%	96.24%	96.50%	96.69%	96.79%	96.95%	97.14%
F1-2	30.77%	76.18%	94.20%	95.21%	95.90%	96.29%	96.60%	96.81%	97.06%	97.21%	97.28%	97.42%	97.54%
POF-1	0.04%	19.80%	83.13%	94.19%	95.25%	95.66%	96.06%	96.25%	96.51%	96.68%	96.80%	96.90%	97.06%
POF-2	0.47%	33.22%	86.61%	94.23%	95.29%	95.69%	96.03%	96.28%	96.58%	96.76%	96.87%	96.98%	97.11%
**Trans**													
**Sample name**	**1kb**	**2kb**	**5kb**	**10kb**	**20kb**	**30kb**	**40kb**	**50kb**	**60kb**	**70kb**	**80kb**	**90kb**	**100kb**
SWF-1	0.01%	0.05%	26.53%	86.89%	95.31%	95.83%	96.24%	96.53%	96.78%	97.04%	97.15%	97.30%	97.43%
SWF-2	0.03%	2.70%	54.90%	93.38%	95.85%	96.34%	96.66%	96.94%	97.22%	97.40%	97.49%	97.65%	97.80%
F1-1	0.01%	0.04%	9.48%	58.31%	91.78%	95.47%	96.01%	96.32%	96.64%	96.91%	97.00%	97.18%	97.25%
F1-2	0.83%	15.96%	80.81%	95.26%	96.18%	96.60%	96.93%	97.17%	97.51%	97.64%	97.73%	97.98%	98.08%
POF-1	0.05%	5.81%	66.34%	94.44%	96.04%	96.54%	96.85%	97.11%	97.46%	97.54%	97.63%	97.83%	97.92%
POF-2	0.56%	21.82%	88.01%	95.50%	96.27%	96.72%	97.06%	97.31%	97.55%	97.64%	97.76%	97.97%	98.07%
**Cis + Trans**													
**Sample name**	**1kb**	**2kb**	**5kb**	**10kb**	**20kb**	**30kb**	**40kb**	**50kb**	**60kb**	**70kb**	**80kb**	**90kb**	**100kb**
SWF-1	16.89%	68.07%	93.90%	95.27%	96.01%	96.44%	96.76%	97.03%	97.30%	97.42%	97.60%	97.72%	97.86%
SWF-2	22.71%	72.93%	94.34%	95.47%	96.20%	96.61%	96.93%	97.17%	97.49%	97.59%	97.73%	97.87%	98.01%
F1-1	10.14%	56.84%	92.87%	95.06%	95.85%	96.31%	96.65%	96.94%	97.24%	97.32%	97.50%	97.63%	97.78%
F1-2	54.85%	86.49%	95.12%	95.86%	96.51%	96.93%	97.24%	97.47%	97.84%	97.90%	98.03%	98.28%	98.36%
POF-1	10.01%	66.88%	94.34%	95.62%	96.33%	96.76%	97.06%	97.33%	97.61%	97.69%	97.80%	98.01%	98.14%
POF-2	0.47%	33.22%	86.61%	94.23%	95.29%	96.92%	97.21%	97.45%	97.72%	97.84%	97.97%	98.19%	98.33%

Supplementary Table 4 The resolution of Hi-C contacts with merged biological replicates in each stage.

Sample name	1kb	2kb	5kb	10kb	20kb	30kb	40kb	50kb	60kb	70kb	80kb	90kb	100kb
SWF	51.20%	84.89%	94.98%	95.78%	96.40%	96.78%	97.07%	97.32%	97.59%	97.63%	97.75%	97.91%	97.98%
F1	65.52%	90.61%	95.47%	96.05%	96.64%	96.98%	97.23%	97.50%	97.68%	97.75%	97.87%	98.01%	98.14%
POF	34.14%	77.73%	94.92%	95.72%	96.31%	96.68%	96.98%	97.23%	97.46%	97.56%	97.68%	97.79%	97.93%
