# Supplementary material for: Dynamic transcriptome and chromatin architecture in granulosa cells during chicken folliculogenesis
Source: Nat Commun. 2022 Jan 10;13:131. doi: 10.1038/s41467-021-27800-9 (PMC8748434; doi:10.1038/s41467-021-27800-9)
Supplement: Supplementary file 3 — Description of Additional Supplementary Files [file 41467_2021_27800_MOESM3_ESM.docx]

File name: Supplementary Data 1.

Description: **Sequencing data summary.**

File name: Supplementary Data 2.

Description: **The top 1% highly expressed genes at each stage.**

File name: Supplementary Data 3.

Description: **Genes with temporal changed expression during folliculogenesis.**

File name: Supplementary Data 4.

Description: **Subset of stage-speciﬁc signature genes for GCs at each stage.**

File name: Supplementary Data 5.

Description: **Annotation of PEIs in convergent CTCF-CTCF loops of SWF, F1, and POF.**
