# Supplementary material for: Dynamic transcriptome and chromatin architecture in granulosa cells during chicken folliculogenesis
Source: Nat Commun. 2022 Jan 10;13:131. doi: 10.1038/s41467-021-27800-9 (PMC8748434; doi:10.1038/s41467-021-27800-9)
Supplement: Supplementary file 9 — Reporting Summary [file 41467_2021_27800_MOESM9_ESM.pdf]

nature research

Corresponding author(s): Diyan Li; Mingzhou Li

Last updated by author(s): Nov 26, 2021

Reporting Summary

Nature Research wishes to improve the reproducibility of the work that we publish. This form provides structure for consistency and transparency in reporting. For further information on Nature Research policies, see our Editorial Policies and the Editorial Policy Checklist.

Statistics

For all statistical analyses, confirm that the following items are present in the figure legend, table legend, main text, or Methods section.

n/a	Confirmed	
☐	☒	The exact sample size (n) for each experimental group/condition, given as a discrete number and unit of measurement
☐	☒	A statement on whether measurements were taken from distinct samples or whether the same sample was measured repeatedly
☐	☒	The statistical test(s) used AND whether they are one- or two-sided<br>*Only common tests should be described solely by name; describe more complex techniques in the Methods section.*
☒	☐	A description of all covariates tested
☐	☒	A description of any assumptions or corrections, such as tests of normality and adjustment for multiple comparisons
☐	☒	A full description of the statistical parameters including central tendency (e.g. means) or other basic estimates (e.g. regression coefficient) AND variation (e.g. standard deviation) or associated estimates of uncertainty (e.g. confidence intervals)
☐	☒	For null hypothesis testing, the test statistic (e.g. F, t, r) with confidence intervals, effect sizes, degrees of freedom and P value noted<br>*Give P values as exact values whenever suitable.*
☒	☐	For Bayesian analysis, information on the choice of priors and Markov chain Monte Carlo settings
☐	☒	For hierarchical and complex designs, identification of the appropriate level for tests and full reporting of outcomes
☐	☒	Estimates of effect sizes (e.g. Cohen's d, Pearson's r), indicating how they were calculated

Our web collection on statistics for biologists contains articles on many of the points above.

Software and code

Policy information about availability of computer code

Data collection	All software used in this study for data collection are either commercially available or open source.
Data analysis	HISAT2 (2.1.0), StringTie (v1.3.3), DEseq2, MaSigPro (v 3.12) and Metascape (http://metascape.org/gp/index.html#/main/step1) were used to analysis the data of bulk RNA-seq.<br>Cell Ranger analysis pipeline (v2.1.1) and R package Seurat (v2.2.014) were used to analysis the data of single cell RAN-seq.<br>Juicer pipeline (v 1.5), BWA-mem (v 0.7.15), HiCrep, miniMDS, Fit-Hi-C and PSYCHIC were used to analysis the data of Hi-C.<br>Cutadapt (v 1.9.1), Bowtie2(v 2.2.6), MACS2(v 2.1.1.20160309), Samtools (v 1.3.1), DESeq2 and MEME suite(https://meme-suite.org/meme/doc/meme.html) were used to analysis the data of ATAC-seq.<br>Trimmomatic (v 0.38), BWA (v 0.7.15), Samtools (v 1.3.1), ROSE algorithms and MACS2(v 2.1.1.20160309) were used to analysis the data of Chip-seq.<br>All statistical analyses were performed by two-tailed Student's t-test, Wilcoxon rank-sum test or Mann–Whitney U test using R.<br>All codes and scripts are available on https://github.com/JiamanZhang/Lab_GCs_paper_codes or on https://doi.org/10.5281/zenodo.5677410.

For manuscripts utilizing custom algorithms or software that are central to the research but not yet described in published literature, software must be made available to editors and reviewers. We strongly encourage code deposition in a community repository (e.g. GitHub). See the Nature Research guidelines for submitting code & software for further information.

Data

Policy information about availability of data

All manuscripts must include a data availability statement. This statement should provide the following information, where applicable:

- Accession codes, unique identifiers, or web links for publicly available datasets
- A list of figures that have associated raw data
- A description of any restrictions on data availability

The Hi-C data of granulosa cells (GCs) generated in this study have been deposited in the GEO database under accession codehave been deposited in GEO under accession codes GSE167064. The bulk RNA-seq, single cell RNA-seq, ATAC-seq, and ChIP-seq data generated in this study have been deposited in the GEO database under accession codeRaw and processed data of bulk RNA-seq, single cell RNA-seq, ATAC-seq, and ChIP-seq have been deposited in GEO under accession codes GSE181756. Chicken hearts single cell RNA-seq data was downloaded from GEO database under accession codes GSE149457. Chicken embryonic fibroblasts, chicken immature and mature erythrocytes Hi-C data was downloaded from GEO database under accession codes GSE96037. All other data supporting the findings of this study are available within the article and its Supplementary Information files or from the corresponding author upon reasonable request. A reporting summary for this Article is available as a Supplementary Information file. Source data are provided with this paper.

Field-specific reporting

Please select the one below that is the best fit for your research. If you are not sure, read the appropriate sections before making your selection.

- [x] Life sciences
- [ ] Behavioural & social sciences
- [ ] Ecological, evolutionary & environmental sciences

For a reference copy of the document with all sections, see nature.com/documents/nr-reporting-summary-flat.pdf

Life sciences study design

All studies must disclose on these points even when the disclosure is negative.

Sample size	No prior sample size determination was conducted. All experiments were conducted in 1-6 biological replicates. Statistical testing ensured significant findings.
Data exclusions	No data were excluded from the study.
Replication	The study integrated RNA-seq (10 stages with 6 biological replicates in each stage) , Single cell RNA-seq (3 stages with 1 biological replicate in each stage),Hi-C (3 stages with 2 biological replicates in each stage), ATAC-seq (3 stages with 1 biological replicate in each stage), ChIP-seq (3 stages with 2 biological replicates in each stage).
Randomization	Not relevant to our study since we did not use settings of experiment/control groups.
Blinding	Not relevant to our study since we did not use settings of experiment/control groups.

Reporting for specific materials, systems and methods

We require information from authors about some types of materials, experimental systems and methods used in many studies. Here, indicate whether each material, system or method listed is relevant to your study. If you are not sure if a list item applies to your research, read the appropriate section before selecting a response.

Materials & experimental systems

n/a	Involved in the study	
☐	☒	Antibodies
☒	☐	Eukaryotic cell lines
☒	☐	Palaeontology and archaeology
☐	☒	Animals and other organisms
☒	☐	Human research participants
☒	☐	Clinical data
☒	☐	Dual use research of concern

Methods

n/a	Involved in the study	
☐	☒	ChIP-seq
☒	☐	Flow cytometry
☒	☐	MRI-based neuroimaging

Antibodies

Antibodies used	Antibody used for immunoprecipitation: Rabbit polyclonal anti-Histone H3K27ac (Abcam, Cat. #ab4729), used at 1:100.
Validation	This antibody was extensively validated in previously published papers and by suppliers.

Animals and other organisms

Policy information about studies involving animals; ARRIVE guidelines recommended for reporting animal research

Laboratory animals	Chicken (Gallus gallus domesticus, Luhua hens, female, 31-week-old).
Wild animals	No wild animals used in the study.
Field-collected samples	No field-collected samples used in the study.
Ethics oversight	All animal protocols were approved by the Institutional Animal Care and Use Committee of Sichuan Agricultural University (protocol number B20171910). The methods were carried out in accordance with the approved guidelines.

Note that full information on the approval of the study protocol must also be provided in the manuscript.

ChIP-seq

Data deposition

☒ Confirm that both raw and final processed data have been deposited in a public database such as GEO.

☒ Confirm that you have deposited or provided access to graph files (e.g. BED files) for the called peaks.

Data access links<br>*May remain private before publication.*	ChIP-seq data have been deposited in GEO under accession codes GSE181756.<br>https://www.ncbi.nlm.nih.gov/geo/query/acc.cgi?acc=GSE181756
Files in database submission	IP-SWF-A-H3K27Ac_1.clean.fq.gz<br>IP-SWF-A-H3K27Ac_2.clean.fq.gz<br>IP-SWF-B-H3K27Ac_1.clean.fq.gz<br>IP-SWF-B-H3K27Ac_2.clean.fq.gz<br>IP-F1-A-H3K27Ac_1.clean.fq.gz<br>IP-F1-A-H3K27Ac_2.clean.fq.gz<br>IP-F1-B-H3K27Ac_1.clean.fq.gz<br>IP-F1-B-H3K27Ac_2.clean.fq.gz<br>IP-POF-A-H3K27Ac_1.clean.fq.gz<br>IP-POF-A-H3K27Ac_2.clean.fq.gz<br>IP-POF-B-H3K27Ac_1.clean.fq.gz<br>IP-POF-B-H3K27Ac_2.clean.fq.gz<br>IN-SWF-A_1.clean.fq.gz<br>IN-SWF-A_2.clean.fq.gz<br>IN-SWF-B_1.clean.fq.gz<br>IN-SWF-B_2.clean.fq.gz<br>IN-F1-A_1.clean.fq.gz<br>IN-F1-A_2.clean.fq.gz<br>IN-F1-B_1.clean.fq.gz<br>IN-F1-B_2.clean.fq.gz<br>IN-POF-A_1.clean.fq.gz<br>IN-POF-A_2.clean.fq.gz<br>IN-POF-B_1.clean.fq.gz<br>IN-POF-B_2.clean.fq.gz<br>IP-SWF_AllEnhancers.table.txt<br>IP-F1_AllEnhancers.table.txt<br>IP-POF_AllEnhancers.table.txt
Genome browser session<br>(e.g. UCSC)	No longer applicable.

Methodology

Replicates	3 stages with 2 biological replicates in each stage.
Sequencing depth	967M of total number reads, 687M of uniquely mapped reads, 150bp length of reads.
Antibodies	Antibody used for immunoprecipitation: Rabbit polyclonal anti-Histone H3K27ac (Abcam, Cat. #ab4729), used at 1:100.
Peak calling parameters	macs2 callpeak --SPMR --bdg --verbose=2 --format=BAMPE --gsize=1065365425 --keep-dup=all --qvalue=0.05<br>bwa mem -t 10 -k 32 -M -R<br>bwa index galGal6.fa

Data quality	The peaks were not validated in this paper.
Software	Trimmomatic (v 0.38), BWA (v 0.7.15), Samtools (v 1.3.1), ROSE algorithms and MACS2(v 2.1.1.20160309) were used to analysis the data of Chip-seq.
